# Treatment of influenza virus-related critical illness during continuous renal replacement therapy: take caution with dosing

**DOI:** 10.1186/s13054-020-2813-y

**Published:** 2020-03-03

**Authors:** Patrick M. Honore, Luc Kugener, Sebastien Redant, Rachid Attou, Andrea Gallerani, David De Bels

**Affiliations:** ICU Department, Centre Hospitalier Universitaire Brugmann-Brugmann University Hospital, Place Van Gehuchtenplein, 4, 1020 Brussels, Belgium

In their discussion, Chow et al. state that for patients who cannot tolerate or absorb enteric oseltamivir due to gastric stasis, malabsorption, or other gastrointestinal processes, intravenous peramivir may be an alternative [1]. They advise a single dose of 600 mg via intravenous infusion, given over 15–30 min [1]. The question that arises is the following: what is the dose to be given during continuous renal replacement therapy (CRRT)? According to the manufacturer’s recommendations, the dose should be adjusted in line with the degree of reduction in creatinine clearance [2]. In a recent case report by Dillon et al., a dose of 200 mg daily, the recommended dose for patients with creatinine clearance of 30–49 mL/min, was given to a patient with acute kidney injury (AKI) managed with CRRT [2]. In practice, as the plasma protein unbound fraction approaches 1 (as is the case with peramivir) and the molecular weight of the drug is low (below 20,000 Da), the sieving coefficient can be estimated to be 1 [3]. As a consequence, in this case report, the dosage scheme (200 mg daily) achieved a smaller serum peak concentration, larger volume of distribution, and a shorter half-life, illustrating the significant differences in pharmacokinetic (PK)/pharmacodynamic (PD) target attainment when drugs are administered in the setting of continuous veno-venous hemodiafiltration (CVVHDF) [2]. Scheetz et al. recommend a steady-state concentration of 17,078 ng/mL, which was averaged from peak and trough concentrations in adult patients with normal renal function [4]. Based on this recommendation, the patient reported by Dillon et al. would have required more than 800 mg given the CRRT settings [2, 4]. Doses of peramivir during CRRT vary from 200 to 800 mg per day in various case reports. This huge difference in dose will have a dramatic influence upon PK/PD during CRRT. Peramivir does not seem to possess any of the classic indications for therapeutic drug monitoring (TDM) (i.e., narrow therapeutic index, high interpatient variability, high intrapatient variability) in patients with normal organ function [5]. However, in patients with critical illness requiring extracorporeal therapies, TDM may provide useful information regarding pharmacokinetic variations and pharmacodynamic target attainment in order to avoid severe underdosing and treatment failure [5].

## Data Availability

Not applicable.

## Abbreviations

AKI: Acute kidney injury
CRRT: Continuous renal replacement therapy
CVVHDF: Continuous veno-venous hemodiafiltration
PD: Pharmacodynamic
PK: Pharmacokinetic
TDM: Therapeutic drug monitoring
